# Assessing performance enhancing tools: experiences with the open performance review and appraisal system (OPRAS) and expectations towards payment for performance (P4P) in the public health sector in Tanzania

**DOI:** 10.1186/1744-8603-8-33

**Published:** 2012-09-10

**Authors:** Nils Gunnar Songstad, Ida Lindkvist, Karen Marie Moland, Victor Chimhutu, Astrid Blystad

**Affiliations:** 1 Centre for International Health, University of Bergen, P.O. Box 7804, Bergen 5020, Norway; 2 Chr. Michelsen Institute, P.O. Box 6033, Bedriftssenteret, Bergen 5892, Norway; 3 Department of Health Promotion and Development, University of Bergen, P.O. Box 7807, Bergen 5020, Norway; 4 Department of Public Health and Primary Health Care, University of Bergen, P.O. Box 7804, Bergen 5020, Norway

**Keywords:** Working conditions, Human resources, Rural health services, Tanzania, Motivation, Performance appraisal, Results-based payment

## Abstract

**Background:**

Health workers’ motivation is a key determinant of the quality of health services, and poor motivation has been found to be an obstacle to service delivery in many low-income countries. In order to increase the quality of service delivery in the public sector in Tanzania, the *Open Performance Review and Appraisal System* (OPRAS) has been implemented, and a new results-based payment system, *Payment for performance* (P4P) is introduced in the health sector. This article addresses health workers’ experiences with OPRAS, expectations towards P4P and how lessons learned from OPRAS can assist in the implementation of P4P. The broader aim is to generate knowledge on health workers’ motivation in low-income contexts.

**Methods:**

A qualitative study design has been employed to elicit data on health worker motivation at a general level and in relation to OPRAS and P4P in particular. Focus group discussions (FGDs) and in-depth interviews (IDIs) have been conducted with nursing staff, clinicians and administrators in the public health sector in a rural district in Tanzania. The study has an ethnographic backdrop based on earlier long-term fieldwork in Tanzania.

**Results:**

Health workers evaluated OPRAS and P4P in terms of the benefits experienced or expected from complying with the tools. The study found a general reluctance towards OPRAS as health workers did not see OPRAS as leading to financial gains nor did it provide feedback on performance. Great expectations were expressed towards P4P due to its prospects of topping up salaries, but the links between the two performance enhancing tools were unclear.

**Conclusions:**

Health workers respond to performance enhancing tools based on whether the tools are found appropriate or yield any tangible benefits. The importance placed on salary and allowances forms the setting in which OPRAS operates. The expected addition to the salary through P4P has created a vigorous discourse among health workers attesting to the importance of the salary for motivation. Lessons learned from OPRAS can be utilized in the implementation of P4P and can enhance our knowledge on motivation and performance in the health services in low-income contexts such as Tanzania.

## Introduction

The World Health Organization (WHO) in its 2006 World Health Report *Working together for health* points to health workers’ motivation as an important determinant of the quality of health care. The report defines health worker motivation as “the level of effort and desire to perform well” [1]:71, and emphasizes the importance of increasing motivation to enhance performance. A growing recognition of inadequate quality of health services in sub-Saharan Africa has led to the introduction of new tools aimed at improving health workers’ performance. In Tanzania, the *Open Performance Review and Appraisal System* (OPRAS) has been introduced in the public sector. Alongside OPRAS, a result-based payment system, *Payment for performance* (P4P), is currently run as a pilot in the health sector. OPRAS is implemented in the public sector whereas P4P is for the health sector only. Both OPRAS and P4P can be seen as performance enhancing tools. OPRAS measures performance at the individual level. P4P also targets individual performance but the measure is whether health facilities or management teams have reached the targets. Little or no research has been carried out on the experiences with OPRAS, and we know little about the relation between this tool and motivation and performance. This paper focuses on health workers’ experiences and raises three important questions. First, how do OPRAS and P4P relate to theoretical approaches to motivation? Second, how do these two tools articulate with health workers’ own understanding of motivation? Third, how can the lessons learned from OPRAS offer insights of relevance for the implementation of P4P in Tanzania? The overarching aim is to contribute to debates on health workers’ motivation in low-income contexts.

### Health worker motivation

Motivation in general concerns a person’s reason for carrying out a particular task. It is necessary to distinguish between motivation from external rewards and motivation existing regardless of rewards. Ryan and Deci argue “[t]o be motivated means *to be moved* to do something.” (italics in original) [2]:54. They state that *intrinsic* motivation implies “doing something because it is inherently interesting or enjoyable” [2]:55, and that it is found where work is performed “for the positive experiences associated with exercising and extending ones capacities” [2]:56. Intrinsic motivation is both situational and personal, and may hence vary between individuals as well as from one situation to another. *Extrinsic* motivation refers to “doing something because it leads to a separable outcome.” [2]:55. Extrinsic factors encompass mechanisms expected to encourage a worker to increase her/his efforts in the expectation of some form of reward or in fear of coercion or sanctions, for example reduced salary.

A much used definition of motivation in the health sector is “an individual’s degree of willingness to exert and maintain an effort towards organizational goals” [3]:1255. This definition is based on the assumption that individual behavior can be changed. An influential approach to workplace motivation is formulated through *agency theory* in economics, a theory which postulates that increased alignment between the goals of principal (employer) and the agent (employee) can be achieved if the principal offers rewards to the agent. Eisenhardt points out that agency theory refers to situations where “the desires or goals of the principal and agent conflict” [4]:58. The underlying assumption of the agency theory is that rewards motivate workers to perform better.

### Health sector challenges in Tanzania

In many low-income countries the health workforce is under serious stress from resource constraints, a situation which generates particular challenges in motivating health workers. A range of studies indicate that there are major problems related to health worker motivation in low-income countries [5-8]. Some studies discuss health workers’ motivation in the Tanzanian context [9-12] and other studies link poor performance to low motivation [13-15]. Similar observations have been made in other countries [16]. It is therefore important to identify factors that impact motivation, and the relation between motivation and performance. A systematic review of health workers’ motivation in low-income countries concludes that financial rewards, career development, continuing education, hospital infrastructure, resource availability, hospital management, and recognition are core contributing factors [17]. Health worker motivation thus emerges as a highly complex issue. Gilson et al. state that health workers’ motivation “reflects a range of personal, organizational, and societal factors, including relationships with others, and itself influences many aspects of the provision of health care.” [18]. The health related Millennium Development Goals (MDGs) constitute measurable targets for health sector performance, and reaching the goals has been given high political priority in Tanzania. Improved access to health services of good quality is the overall aim of the *National Health Policy *[19], the *Primary Health Services Development Programme 2007–2017 *[20] and the *Human Resource for Health Strategic Plan *[21]. The common focus of these policies and plans is to improve the quality of services through expanding the number of health facilities and health workers and to increase access to resources. Moreover, there is an increased focus on health workers’ performance. It is partly to this end several performance enhancing tools have been implemented.

### Implementing OPRAS

In Tanzania, the Public Sector Reform Programme (PSRP) aims at improving public sector service delivery. The Open Performance Review and Appraisal System (OPRAS) was introduced in 2004 [21]:11, and replaced a former confidential performance appraisal [22]:12, [23]:21. It is stated about OPRAS that “[t]he introduction of this system aims at improving the quality of public services in Tanzania” [24]:252. OPRAS seeks to improve performance through setting individual goals, measuring the achievement of the goals and providing feedback. It is argued that OPRAS makes up “an integrated system for building a shared vision, understanding and agreement about the *results* to be achieved, and the *approach, deployment, assessment* and *review* of activities for continuous improvement in standards of service delivery” (italics in original) [24]:252. The principle of OPRAS is that the employee sets targets in consultation with the supervisor. After six months, the achievements are to be evaluated and after 12 months the achievements of the past year are evaluated and the supervisor and employee come to an agreement on the performance to be recorded in OPRAS.

In Tanzania, OPRAS was introduced in the wake of the extensive Public Sector Reform Programme (PSRP) and strategies vested in the New Public Management paradigm following in the wake of the extensive structural adjustment policies. The New Public Management approach has increasingly been adopted in low-income countries [25], a strong manifestation of processes of globalization. OPRAS plays an important role in the improvement of service delivery and the *Public Service Act* defines OPRAS as a compulsory performance appraisal in the public sector.

### Introducing results-based payment

Results-based payment has been introduced in a number of countries, and is marketed as a new and innovative solution to combat low health worker motivation. A general definition of results-based payment is the “[t]ransfer of money or material goods conditional on taking a measurable action or achieving a predetermined performance target” [26]:4. Trisolini argues that results-based payment can be seen as a direct response to the principal-agent problem “by providing objective quality measures” and by “linking payment to improvements in performance.” [27]:80.

In Tanzania, Payment for performance (P4P) has replaced a system of extra allowances to leadership positions, the Selected Accelerated Salary Enhancement (SASE) [28]:6. Donors have been instrumental in introducing P4P in Tanzania and the initiative to introduce P4P has taken place outside the established collaboration between donors who support the Health Basket Fund. Morgan and Eichler report that the Government of Tanzania “decided to implement a P4P scheme without the endorsement of the country’s health sector development partners” [29]. Two important official documents on P4P were issued by the Ministry of Health and Social Welfare (MoHSW) in 2008: the *Payment for Performance Strategy 2008–2015 *[30] and the *Implementation Guideline Payment for Performance *[31]. It was stated that “the results-based bonus system constitutes an important programme strategy which will increase the ability to unleash the energy and creativity needed to address local challenges.” [31]:1, hence indicating motivational as well as performance dimensions of P4P. The introduction of P4P in Tanzania takes place in the context of the *Health Sector Strategic Plan III* which states that P4P will improve both motivation and productivity of health workers [32]:5.

Rewarding health facilities for achieving set targets requires very good tools to measure their performance. An appraisal study of the planned P4P clearly pointed out that the state of the Health Management Information System (HMIS) was far from being of adequate quality [33]:14. Similar concerns had been expressed by two feasibility studies preceding the implementation documents [28,34]. These challenges made donors concerned that the original plans for P4P would be difficult to implement, and the appraisal study warned against “harmful effects if the programme is introduced too rapidly without careful management” [33]:6. Because of the concerns voiced, the process of implementing P4P was halted in 2009 [29]:24,25. Health workers in the study district had however received extensive information about the initial P4P plans before they later were informed that the plans had been halted. Despite the official halt, a P4P scheme following the initial plans is reported to have been implemented in Mvomero District, Morogoro Region [35] as well as in some other districts [29]:25. In 2010, a new P4P plan far more detailed in terms of indicators and procedures for verification of the data as well as in terms of procedures for payment of P4P bonuses was presented [36]. In contrast to the previous plan of a simultaneous roll-out of P4P throughout Tanzania Mainland, the new plan is implemented as a pilot in the Coast Region. The current situation of implementation of P4P alongside OPRAS and a Health Management Information System with shortcomings warrants a study of how both OPRAS and P4P are conceptualized by health workers. The broader aim is to enhance the understanding of work related motivation and performance in the health sector in a low-income setting.

## Methods

### Study setting

The research was carried out in Mbulu District in Manyara Region in northern Tanzania. In terms of livelihood and socio-economic conditions, the district is a typical rural district in Tanzania. The public health facilities in the district comprise a district hospital, two rural health centres and 19 dispensaries. In addition to the public health services there is a large church-run hospital located in the district, and the population thus enjoys a relatively good coverage of health services. There is little indication this has any influence on the working conditions in the public health sector. There is a shortage of all cadres of trained health workers but a surplus of untrained medical attendants in the district. The shortage of qualified staff is most pronounced at health centre- and dispensary levels. Many nurse positions in these health facilities are filled by medical attendants, a cadre with very little formal education. At the time of the data collection there was no fully trained Medical Officer (MD) in the public health services.

### Data collection

A qualitative study design was chosen to explore health workers’ perception of motivation, their experiences with OPRAS and expectations towards P4P. The two main methods for collection of data in our study were *in-depth interviews* (IDIs) and *focus group discussions* (FGDs). The IDIs and FGDs were carried out at locations providing the necessary privacy to allow interviewees to speak freely. The first author and several of the co-authors speak Swahili, the national language in Tanzania, and have been engaged in long term ethnographic fieldwork in Tanzania. This competence serves as a backdrop to the present study.

The formative phase of the research took place in April and May 2007 and the main research topics to be pursued were identified at this point. The bulk of the data were collected during the second phase between October 2007 and February 2008. More targeted IDIs in the public health sector were carried out in May 2009 and additional FGDs with a particular focus on OPRAS and P4P were carried out in May 2010. Interview guides and topic guides were employed in all IDIs and FGDs, and were applied with great flexibility to allow for time to be spent on important or emerging issues. The focus on OPRAS and P4P was increased in the IDI and FGDs during the course of data collection. Documents collected during the course of the research period were also systematically reviewed. Government documents in English and Swahili provided useful data on relevant legislation and government policies related to the human resource situation and to challenges in the health sector. Annual plans and reports from health facilities in the study district also provided useful information to understand the local human resource situation.

At the time of data collection the OPRAS had been in use for several years and P4P had been introduced to the health workers in the study district. A general lack of transparency related to issues such as salaries, allowances and promotion policies led to difficulties in gaining access to reliable information on both OPRAS and P4P. The opportunity to express experiences and expectations with the performance enhancing tools was welcomed, but the negative aspects may possibly have been exaggerated.

### Research participants

Research participants were recruited to cover diverse categories of public sector health workers, different types of health facilities as well as both central and rural parts of the district. The data comprise 20 IDIs with various cadres of health workers and five administrators, four of whom were Council Health Management Team (CHMT) members and one was a manager in the district administration. Six FGDs were carried out at the district hospital with a total of 29 participants. The IDIs and FGDs were carried out in Swahili by the first author with the assistance of a Tanzanian research assistant (Tables 1 and 2).

**Table 1:** Overview of IDIs with health workers (excl. administrators)

	**Dispensary**	**District hospital**
Medical attendants	2	2
Nursing staff	4	4
Clinical Officer (CO)	4	2
Assistant Medical Officer (AMO)	0	2

**Table 2:** Overview of FGDs

	**Participants**	**Men**	**Women**
Nursing staff	5	0	5
Nursing staff	6	0	6
Clinicians (AMO/CO)	6	6	0
Nursing staff	5	0	5
Nursing staff	3	0	3
Nursing staff	4	0	4

### Data analysis

All the IDIs and FGDs were tape recorded, transcribed and translated to English by research assistants. Thorough checks on the translation from Swahili to English were carried by the first author to reduce the risk of misinterpretation of the data. Audio files, transcripts of audio files and research notes were managed by NVivo 8 and NVivo 9 for systematic data management and analysis. The material was subject to a thorough review and coding of the content. The data analysis followed the four steps suggested by Malterud [37]:111. The first step is to identify the broader themes present in the data. The second step is to assign codes to the data. The third step is to develop a structured set of codes. The fourth step requires reflection to systematise the codes into larger units, or categories.

### Research ethics

The study is part of a collaborative research venture funded by the Research Council of Norway entitled *Strengthening human resources for health: A study of health worker availability and performance in Tanzania.* The National Institute of Medical Research (NIMR) in Tanzania granted ethical clearance for the project (ref. NIMR/HQ/r.8a/Vol. IX/433). Permission was also obtained from the Tanzania Commission for Science and Technology (COSTECH) (2007-59-CC-2006-193, with extensions 2008-181-ER-2006-193 and 2009-250-ER-2006-193). A letter from the Regional Administrative Secretary was presented to the administration in the study district. All informants participating in the IDIs and FGDs received information about the research and about the voluntary nature of the research both verbally and in writing before signing a consent form, complying with the regulations of the Tanzania National Health Research Forum [38]:25-30.

## Results

In this section we will explore what health workers in the study district held as important for motivation, their experiences with OPRAS and expectations towards P4P.

### Perceptions of motivation

The interviewed health workers used two Swahili terms for motivation. The most frequently used term was *motisha*, denoting the employer’s effort to motivate staff through offering incentives. The other term used to describe motivation was *hamasa*, a term that can be translated as ‘determination for work’. One Nursing Officer made a clear distinction between being motivated because of working conditions with reference to *motisha* and being motivated as a calling, or *hamasa*:

This work requires a calling. If one bases the work on the salary alone, then one would say ‘why worry with such little pay?’ But if the person is committed whole-heartedly to the serve the patients, then he/she can perform the work well even though the payment is poor. (Nursing Officer, district hospital, IDI)

Health workers readily acknowledged that work effort varies and they pointed out a number of issues they consider motivating for effort at the workplace.

#### Salary and allowances

All the interviewed health workers emphasized that a decent salary is very important for their motivation. It was consistently argued that the basic salary is too low to make ends meet. One Assistant Medical Officer (AMO) explained that:

If people really want us to provide good health services, the health workers should be paid a reasonable salary, enough to meet the basic important requirements, at least enough to be able to send children to school and have decent clothing. (AMO, district hospital, IDI)

The role of financial incentives was also explained:

If you get something in addition to the salary you will maintain a high level of performance and have more zeal. (AMO, district hospital, IDI)

The general experience of receiving a low salary generated discontent and demands for additions to the salary. A recurring comment was that a low salary and a high workload make staff demotivated. A nurse commented on the importance of additional payment for working morale:

When one gets an extra income then certainly one becomes motivated and performs work better. Working continuously without any improvement of the income and with an increasing workload, one becomes demotivated, and the working morale is lost or reduced. (Nurse, district hospital, FGD)

In addition to the concern about the salary, substantial emphasis was placed on the allowances, in particular the entitled allowance when attending seminars, short courses or workshops. One Clinical Officer (CO) explained:

When we are being trained in seminars we get good allowances. Such an income could not be achieved in a whole month of work, but it can be obtained in, say, one week of seminar. (CO, district hospital, IDI)

The emphasis placed on the financial aspects of working conditions, for example the salary level and the extra allowances attest to the importance of *motisha* as vital for motivation. Health workers however also emphasized non-financial aspects of working conditions as vital for motivation.

#### Recognition of good performance

Recognition of good performance was pointed out as important for motivation. One AMO explained:

If a leader stands up in a general meeting of all staff and announces that a certain group of people have done very well, the people praised will become motivated. (AMO, district hospital, IDI)

A similar argument was put forward by one nurse:

Even a simple letter showing that you have done well, or even an expression of congratulations for the good work would really give a person encouragement to work. (Nurse, district hospital, FGD)

Health workers claimed that they do not receive regular feedback on the work they perform and held this out as a serious shortcoming of the workplace management. Health workers however pointed out a few exceptions, one being the annual 1st May, *Workers’ Day*, celebration with the announcement of *Mfanyakazi Bora*, - the ‘worker of the year’. In addition to the public recognition of having performed well, the awardees receive a gift in cash or in kind. Several of the interviewed health workers had previous experience of being selected as the worker of the year. One CO explained:

If one of the staff is selected here, his colleagues will work very hard so that they can become selected the next time. (CO, dispensary, IDI)

A Council Health Management Team (CHMT) member who explained that he had received a letter from the District Executive Director (DED) thanking him for his effort and that this encouraged him to perform better and how it gave him the determination to work better than before. Receiving recognition from the patients was also held out as a motivating factor. One nurse explained:

The gratitude from patients is also giving me determination because it is an indicator that my performance at work is good and helpful to the patients. (Nurse, district hospital, FGD).

The interviewed health workers consistently pointed out the importance of receiving feedback on performance and also pointed out the importance of supervision. The examples above attest to the importance health workers place of being recognised for the work they perform.

### Perceptions of OPRAS

The interviewed health workers had gained experience over several years of filling in the OPRAS form. The overall picture emerging from the data was a high degree of skepticism towards OPRAS. The perceptions of OPRAS however ranged from seeing it as being of little value to perceiving it as a positive development. The interviewed health workers, regardless of their stance towards OPRAS, referred to it as a system in which individual goals are set and performance measured. However, the interviewed health workers raised a series of concerns about whether OPRAS is reaching its aim, and to what extent it is appropriate in the health sector.

#### Number of patients vs. quality care

Many health workers expressed concerns about measuring performance through OPRAS in a setting of shortage of resources. A recurring argument was that the shortage of resources at the workplace makes it very difficult for health workers to reach their targets. It was also argued that the measurements of performance in OPRAS are of little relevance and help in the health sector.

The one who has attended 10 patients and correctly diagnosed and treated them well has performed better than the one who has seen 20 patients and made mistakes or prescribed the wrong medication. The important issue is not attending many patients but providing correct treatment. (CO, district hospital, IDI).

One Assistant Medical Officer expressed clear skepticism towards setting targets in terms of number of patients in OPRAS stated:

I am supposed to see or attend 300 patients. What if people in the communities hear that this is my target? I don’t know how they would feel. It could mean that we are praying for them to get sick so that we can achieve our targets. I don’t get the logic behind that. Should we go to the churches and mosques praying to get more patients? (AMO, district hospital, IDI).

A Clinical Officer raised a similar concern:

On the side of a clinician it is difficult to aim at treating a certain number of patients. We don’t want people to become sick. (CO, district hospital, IDI).

The principle of using numbers of patients as an indicator for performing well was hence met with skepticism. Health workers repeatedly pointed out that the optimal situation should be a low number of patients receiving proper treatment.

#### Missing feedback

OPRAS is intended to evaluate performance and to provide feedback to the employee. In practice, however, OPRAS appeared not be used for providing feedback.

We would request that feedback is given to us, because it is a difficult process to fill in the OPRAS form. You do this work and you don’t get any feedback. It becomes tiresome and meaningless, wasting our time and effort almost for nothing, feedback is very much needed! (Nurse, district hospital, FGD).

A Clinical Officer brought up the challenge of verifying whether targets in OPRAS are achieved:

At the end of the day I don’t think that there is any person coming to inspect or to verify that you attend the 200 children you said you would treat. (CO, district hospital, IDI).

Experiences with OPRAS indicate that many of the interviewed health workers do not see the benefits of the system. One AMO explained:

Up to now there is nothing coming out of filling in the OPRAS form. It is just like an order. After every six months they tell us to fill it in and again. It is just a routine but it has no meaning. (AMO, district hospital, IDI).

The large majority of health workers interviewed expressed great skepticism towards OPRAS and furthermore explained that they have little knowledge about the use of the information collected through OPRAS. The degree of reluctance towards complying with OPRAS indicates that dissemination of information on the performance appraisal and providing feedback to health workers has been insufficient.

#### Openness and opportunities

Some health workers did mention benefits of OPRAS and it was repeatedly pointed out that OPRAS is a better system than the earlier confidential assessment. One nurse explained:

I think OPRAS is better because it is a more open system. You can express or defend yourself, or if you find that you may have been badly treated you have the chance to complain. (Nurse, district hospital, FGD).

Other health workers expressed that OPRAS was an important tool guiding them at work.

It is possible for me to adjust myself at work. Where I have been reluctant or where I have been negligent, the in-charge may warn me before things get worse. (Nurse, district hospital, FGD).

The most concrete examples of employees’ positive evaluation of OPRAS related to promotions. In the latter part of the data collection for this study some of the interviewed health workers reported to have received the expected promotions. One nurse expressed her experiences with OPRAS in relation to promotions as follows:

We were told that a worker cannot be promoted without OPRAS, and that only when one has properly filled in the OPRAS form can promotion be considered. (Nurse, district hospital, FGD).

Members of the Council Health Management Team (CHMT) and the Human Resource Officer in the district generally praised OPRAS, and explained that it provides an opportunity to evaluate the performance of staff, but remained vague as to its relation to promotions.

### Mid-level managers’ experiences with OPRAS

Managers at sections or departments in the health facilities are responsible for working with staff members in order to set and evaluate the individual goals in OPRAS. Health workers reported to have job descriptions but these descriptions appeared not to be actively used to guide the work or to direct effort to tasks important for the quality of health services. OPRAS was perceived by the managers as an attempt to operationalise the job descriptions but a manager in the district administration noted that there was a paucity of knowledge about the ways in which OPRAS is expected to operate:

The employees do not yet understand OPRAS, therefore it is giving them problems in filling in the forms properly. Or you may find that a person is unable to estimate the targets. Thus they may fill in the forms with inaccuracy, while others simply evade it. (Manager in district administration, IDI).

Also the managers brought up the challenges of measuring performance:

The weakness of OPRAS is that you find that an employee has a job description in which its target cannot be measure or evaluated. (Manager in district administration, IDI).

The mid-level managers reflected well on the dynamics of OPRAS on OPRAS, on both its weaknesses and opportunities. They recognized health workers reluctance towards OPRAS as well as the structural problems of resource shortages and the challenges this may cause in implementing OPRAS.

### Expectations towards P4P and its link to OPRAS

The planned P4P in Tanzania is based on measuring performance through assessing whether quantifiable targets have been met at the health facility level. As explained above P4P was not yet implemented in the study area at the time of the data collection. The informants nonetheless had substantial expectations towards P4P due to the information that had been disseminated on the possibilities of additional payment. Health workers expressed positive expectations towards P4P, and perceived that the forthcoming financial incentives would enhance their motivation for work. One nurse explained:

It is encouraging because it is something extra, an extra income on top of the salary. It gives hope to workers that one may get an extra income that can help the family, for example to pay for school fees. (Nurse, district hospital, FGD).

The interviewed health workers however expressed varying degrees of knowledge about the background and rationale for P4P. They generally saw OPRAS and P4P to be two connected systems.

When they introduced P4P they emphasized that we should properly fill in OPRAS so that we could be given the P4P payments. (Nurse, district hospital, FGD).

The initial plans for P4P were however halted, much to the disappointment of the health workers who clearly expected to receive additional payment when meeting their targets. The high expectations coupled with limited information created much confusion about the aims of both OPRAS and P4P, and the possible connections between the two.

## Discussion

Health workers’ motivation is a vital determinant of the quality of the health services [1,39]. A general and accepted premise is that motivated health workers work with an effort to provide the best possible health services under the prevailing resource situation. In settings where the availability of resources is severely compromised, health workers’ motivation becomes particularly vital for performance. Both OPRAS and P4P are tools that aim at improving performance, but do not address health workers’ motivation per se. However, health workers’ experiences with OPRAS and perceptions of P4P can increase our understanding of important dimensions of health workers’ motivation in a low-income context. In the following we will discuss how OPRAS and P4P articulate with health workers’ perceptions of motivation and what has emerged as key challenges in the implementation of the two performance enhancing tools.

### The importance of intrinsic motivation

As presented in introduction, the *Self-determination theory* distinguishes between *intrinsic* and *extrinsic* motivation [40]. Both intrinsic motivation and extrinsic motivation are important for performance of workplace tasks. Ryan and Deci argue that motivation can be conceptualized as a continuum ranging from a motivation to intrinsic motivation [2]:61, [41]:72. A generally accepted assumption is that health workers, as any other employee, are placed between the two extremes of this continuum. The *Self-determination theory* assumes that satisfaction of basic psychological needs represents the underlying mechanisms of intrinsic motivation. It focuses on *competence*, *relatedness* and *autonomy* as key aspects of intrinsic motivation. Of particular relevance in the study of workplace motivation is competence, and Deci and Ryan argue that people “seek challenges that are suited to their competencies, that are neither too easy nor too difficult” [40]:32-33. Relatedness refers to interaction with other people while autonomy refers to being ‘causal agents’ in one’s own life. Thus a working environment where a staff member handles the work tasks well, has good relations with colleagues and superiors, and where the staff member can influence the workplace will be conducive to increased intrinsic motivation. Intrinsic motivation does not remain constant, but is influenced a number of factors. If health workers experience difficult working conditions in terms of shortage of resources or inadequate human resource management one or more of the three elements of intrinsic motivation may be compromised. Performance in the health sector encompasses more than what is possible to measure through numerical indicators. Thus it is very important to ensure that health workers are motivated to exert effort also in workplace tasks not measured. Intrinsic motivation has to be upheld as it is in flux and exists in the nexus between a person and a task.

### OPRAS and P4P in relation to intrinsic and extrinsic motivation

Both OPRAS and P4P can be seen as attempts to align health workers’ goals to the goals of the employer. Both tools are based on the principle that work performance can be measured. P4P offers financial rewards to health facilities having achieved set goals. OPRAS does not offer concrete financial rewards but measuring individual performance is intended to be the basis for indirect financial rewards, for example through promotions or selection for further training. Our study indicates that health workers have great expectations towards the forthcoming P4P, but also that they are reluctant towards OPRAS. P4P appears to be praised because of the expectations of additional payment, whereas OPRAS enjoys little legitimacy partly because health workers claim that the system does not offer any tangible benefits.

It is important to address the socio-economic contexts when attempting to unravel the dynamics at work in health workers’ responses to OPRAS and P4P. Justice importantly calls for approaches that address “cultural settings in which policies and plans filter down through stages of implementation to interact with cultures at the local level” [42]:330. It is argued that performance appraisals need to carefully address the organizational context in which they are to be implemented [43]:883 and the same holds true for other performance enhancing tools. In order to understand responses to and compliance with the OPRAS and the perceptions of P4P, it is important to look closer at the significance of the salary in the discourse on working conditions. In a setting where a health worker may be the only breadwinner in a family with a large number of dependants, the salary and any additions to the basic salary become very important. Health workers interviewed in our study consistently argued that their salary was too low to make ends meet and that children’s school fees in particular constitute a financial hurdle. Recent studies from Tanzania exploring what health workers find important to motivate them at the workplace emphasise the financial part of the working conditions [44,45]. Other studies from Tanzania and other sub-Saharan countries have also found that health workers strongly emphasise the role of the salary and any additions to the salary for their motivation to work. The practice of ‘chasing allowances’ in many countries in sub-Saharan Africa [46], i.e. to try to maximise the amount of allowances earned through participation in seminars or workshops must be understood within a context of experiences of a low salary level. Health workers thus welcome the possibility to earn extra bonuses through P4P and likewise express reluctance towards OPRAS not providing direct financial gains.

National health policies setting the agenda at the local level are informed by global strategies and policies. Both OPRAS and P4P have been implemented through a top-down process and donors have been instrumental in implementing P4P. National and international policies have substantial impact on the local health system. Gilson et al. argue however that health policies and systems are “understood to be **constructed and brought alive** by social actors through the meaning they attach to (their interpretations of) their experiences.” (emphasis in original) [18]. The perspectives of social actors who construct meaning around their workplace experiences help us understand how motivation is a phenomenon shaped in the local context. Our study found *hamasa* and *motisha* to be two central concepts relating to motivation. Health workers referred to *hamasa* as a calling, or inner motivation or determination for work. The experience of finding the work meaningful through good collaboration with supervisors, as well as the patients, clearly seem to increase the commitment towards work and thus stand out as an important aspect of *hamasa*. At the same time, difficult working in terms of resource shortage seem to have a negative impact on intrinsic motivation and this attests to the argument that intrinsic motivation exists in the nexus between a person and a task. *Motisha* is a concept referring to various financial incentives. Attending seminars and workshops with the entitled allowance was perceived as part of the *motisha* concept. The importance placed on external rewards through the coming P4P and the lack of tangible rewards through OPRAS clearly relates to health workers’ perception of what is required to increase extrinsic motivation. P4P thus seems to be firmly based in the domain of *motisha* and health workers evaluate the system based on the benefits it was expected to provide. OPRAS as it is not seen as providing direct tangible incentives is thus not located in the *motisha* domain, neither is it located in the *hamasa* domain.

Central to enhancing intrinsic motivation is recognition of good performance and the experience of playing an important role at the workplace. Recognition is in principle an external factor but it not conceptualized as *motisha* in the health workers’ discourse. Health workers however clearly expressed that recognition is a very important factor to increase determination, to give encouragement and to make the work more enjoyable. Supportive leadership can make a staff member feel valued and important at the workplace, and is thus an example of a mechanism that may nourish intrinsic motivation. The *Self-Determination theory* and its focus on competence, relatedness, and autonomy attest to the importance of recognition for motivation. Ryan discusses the dynamics of motivation and the processes of internalisation of extrinsic factors, emphasizing the importance of a conducive environment and argues that it is “an ongoing process that is influenced by social-contextual conditions in the immediate environment” [47]:420. Ryan and Deci argue that “[c]ontexts supportive of autonomy, competence, and relatedness were found to foster greater internalization and integration than contexts that thwart satisfaction of these needs” [41]:76. The lack of feedback related to the goals set in OPRAS thus implies that important opportunities for increasing intrinsic motivation are not utilized. We may sum up the dynamics at work affecting intrinsic and extrinsic motivation in the following way:

### Challenges in implementing OPRAS

Performance appraisals are intended to be used for decision making related to promotions, access to training, decisions on salary and termination of contracts. Thus, a performance appraisal is a constituent part of the wider human resource management. The Council Health Management Team members and staff at the office of the Human Resource Officer claimed that OPRAS is intended to play a role in assessing individual performance and for example to determine eligibility for further training and promotions. This dimension of OPRAS did however not emerge as clear to the interviewed health workers. OPRAS could ideally be a system to provide feedback on work related efforts, but this possibility appeared not to be utilized. The negligence in filling in the OPRAS form thus appears to be closely linked to health workers not seeing any benefits in terms of tangible rewards or concrete feedback on work. Collecting and storing information on individual performance without providing feedback seemed to undermine the trust in OPRAS. Moreover, health workers question the way performance in the health sector is measured through OPRAS and it was warned against measuring performance through indicators such as counting number of patients attended to. Health workers argued that setting quantifiable targets may not be appropriate because of the risk of a focus on quantity over quality. It was repeatedly stated that proper examination of each patient should be the goal rather than the goals set in the OPRAS form.

The Public Sector Reform Programme (PSRP) represents the context for the design and implementation of OPRAS and other administrative tools aimed at ensuring better management of resources, including human resources. Issa argues that “[s]ynergy between different initiatives has been observed both between the different HR tools and the processes they are to improve” [48]:49. However, an external evaluation of the health sector in Tanzania states that OPRAS “is clearly an improvement over the confidential reports, made by the superior alone. But without clear job descriptions and individual targets, there is no objective basis for assessment.” [49]:85. The President’s Office – Public Service Management states that OPRAS is not linked to sanctions or rewards, and that performance targets are vague or too easy to meet [22]:29. Another record from the same office comments that “its standardised nature and inapplicability to certain job groups; a perception that it does not link to improvements in rewards; and the difficulty of undertaking objective assessments in situations where possibilities of collusion might be prevalent.” [50]. Moreover, it is reported that OPRAS is implemented without coordination with the need for training of staff [51]:4. The challenges in the implementation of OPRAS are thus also acknowledged at the central government level in Tanzania.

### Lessons learned from OPRAS important for the implementation of P4P

The introduction of Payment for performance (P4P) in the context of a performance appraisal system not working as intended should initiate an evaluation of OPRAS and a thorough discussion of how to coordinate the two systems. No official document outlining the P4P modalities, neither the initial plans [30,31], nor the revised pilot [36] carry any references to OPRAS. The same observation is made of the preceding feasibility studies of P4P [28,34]. Only the appraisal study of P4P points out the importance of ensuring links between OPRAS and P4P [33]:19. As both instruments aim at enhancing worker performance, it is noteworthy that the introduction of P4P alongside OPRAS takes place with no mentioning of the dynamics between the two programmes.

Evidence from economic literature indicates that employers can effectively improve performance by offering rewards increasing extrinsic motivation. Concerns have been raised that strategies increasing extrinsic motivation through rewarding easily measureable indicators may reduce the intrinsic motivation. Deci found that “when money was used as an external award, intrinsic motivation tended to decrease” [52]:105. It is argued that introducing incentives aimed at increasing extrinsic performance may have a negative impact on intrinsic motivation [26]:2. The risk of a selective focus on tasks being rewarded has been pointed out by e.g. Rosenthal and Frank who argue that “agents behave strategically, and pay-for-performance programs need to be designed carefully” [53]:153. Golden and Sloan argue that “extrinsic motivation may crowd out the effort that would have been forthcoming in the absence of extrinsic motivators” [54]:296. If only what is measurable is rewarded, this creates a problem in the health sector as not all tasks conducted by health workers are easily measured. If important tasks are not rewarded because they cannot be measured, these tasks may be ignored and more attention paid to tasks for which the health worker is rewarded. Holmstrom and Milgrom argue that incentives “direct the allocation of the agents’ attention *among* their various duties.” (italics in original) [55]:25. A recent study from Rwanda focusing on the effectiveness of results-based payment schemes, found most effect on services that had the highest payment rates and on the services that needed the least effort from the health worker [56]. A review study of results-based payment found that the effectiveness of such programs is highly variable [57]. In Tanzania, the effect of P4P on activities not rewarded remains highly uncertain.

**Figure 1:**
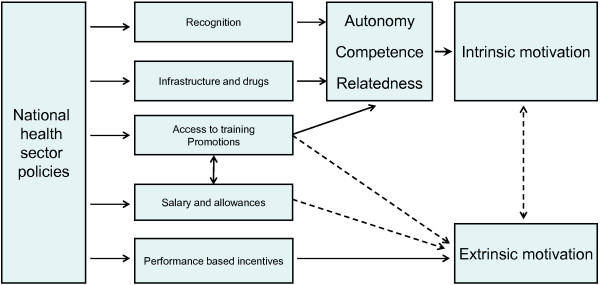
Impact of working conditions on intrinsic and extrinsic motivation.

Our study has indicated that health workers in a resource constrained setting place substantial emphasis on extrinsic motivation, in particular financial incentives on top of the basic salary. Despite increased salary in the health sector in Tanzania in recent years, health workers strongly express dissatisfaction with the working conditions and the financial return on the work. This creates both opportunities and challenges. Health workers may respond positively to financial incentives offered under P4P, and may thus improve performance on work tasks rewarded. This may lead to a higher degree of alignment between the employer’s expectations and the employees’ performance which in turn may lead to increased quality of health services. However, the nature of P4P is likely to further accentuate the emphasis placed on financial incentives at the expense of intrinsic motivation. This should raise concerns about the current strategies and their sustainability in efforts to improve health worker performance in Tanzania (Figure 1).

## Conclusion

The subjects of the performance enhancing tools, the health workers, respond and comply based on their interpretation of whether the tools are found appropriate or whether they yield any benefits. The findings of our study indicate that OPRAS does not work as intended due to its modalities of measuring performance, the poor implementation of the feedback mechanism and health workers’ experience of not seeing any tangible benefits of OPRAS. The expected additions to the basic salary through the coming P4P scheme have created a vigorous discourse among health workers attesting to the importance of the salary level and allowances for motivation. The implementation of OPRAS and the coordination between OPRAS and P4P needs urgent attention to ensure that the lessons learned from OPRAS can be drawn upon to improve approaches to enhance health worker motivation and performance in the health services in Tanzania and beyond.

## Competing interests

The authors declare that they have no competing interests.

## Authors’ contributions

NGS planned and designed the study under the supervision of AB. The data collection was carried out by NGS with assistance of a research assistant. The initial data analysis and development of the first drafts of the article were carried out by NGS under the supervision of AB. IL, KMM and VC contributed substantially to subsequent versions of the paper and critically revised the manuscript. All the authors read and approved the final manuscript.
